# Efferocytosis: the resolution of inflammation in cardiovascular and cerebrovascular disease

**DOI:** 10.3389/fimmu.2024.1485222

**Published:** 2024-11-26

**Authors:** Bingtao Zhang, Yan Zou, Zixuan Yuan, Kun Jiang, Zhaoxiang Zhang, Shujuan Chen, Xiaoming Zhou, Qi Wu, Xin Zhang

**Affiliations:** ^1^ Department of Neurosurgery, Nanjing Jinling Hospital, Affiliated Hospital of Medical School, Nanjing University, Nanjing, China; ^2^ Department of Neurosurgery, Jiangsu Province Hospital of Chinese Medicine, Affiliated Hospital of Nanjing University of Chinese Medicine, Nanjing, China

**Keywords:** efferocytosis, phagocytosis, inflammation, cardiovascular disease, cerebrovascular disease

## Abstract

Cardiovascular and cerebrovascular diseases have surpassed cancer as significant global health challenges, which mainly include atherosclerosis, myocardial infarction, hemorrhagic stroke and ischemia stroke. The inflammatory response immediately following these diseases profoundly impacts patient prognosis and recovery. Efficient resolution of inflammation is crucial not only for halting the inflammatory process but also for restoring tissue homeostasis. Efferocytosis, the phagocytic clearance of dying cells by phagocytes, especially microglia and macrophages, plays a critical role in this resolution process. Upon tissue injury, phagocytes are recruited to the site of damage where they engulf and clear dying cells through efferocytosis. Efferocytosis suppresses the secretion of pro-inflammatory cytokines, stimulates the production of anti-inflammatory cytokines, modulates the phenotype of microglia and macrophages, accelerates the resolution of inflammation, and promotes tissue repair. It involves three main stages: recognition, engulfment, and degradation of dying cells. Optimal removal of apoptotic cargo by phagocytes requires finely tuned machinery and associated modifications. Key molecules in efferocytosis, such as ‘Find-me signals’, ‘Eat-me signals’, and ‘Don’t eat-me signals’, have been shown to enhance efferocytosis following cardiovascular and cerebrovascular diseases. Moreover, various additional molecules, pathways, and mitochondrial metabolic processes have been identified to enhance prognosis and outcomes via efferocytosis in diverse experimental models. Impaired efferocytosis can lead to inflammation-associated pathologies and prolonged recovery periods. Therefore, this review consolidates current understanding of efferocytosis mechanisms and its application in cardiovascular and cerebrovascular diseases, proposing future research directions.

## 1 Introduction

Cell death and turnover are continuous processes essential for maintaining physiological equilibrium across all human organs. Even under healthy conditions, the human body undergoes the turnover of over one million cells per second primarily through programmed cell death, known as apoptosis. Efferocytosis, the phagocytic removal and recycling of dying cells by specialized phagocytes at specific sites, is pivotal in this cellular clearance process (1). Furthermore, efferocytosis assumes critical significance in various pathophysiological contexts such as acute myocardial infarction and transient middle cerebral artery occlusion, prevalent in cardiovascular and cerebrovascular diseases (2–4). Upon injury, extensive cell death leads to the release of damage-associated molecular patterns (DAMPs), triggering localized inflammatory responses (5, 6). Concurrently, dying cells release signals that recruit phagocytes (7) to the injury site, facilitating the clearance of dying cells and DAMPs to mitigate inflammation and restore homeostasis (8). Following the engulfment and recycling of dying cells, phagocytes contribute to the resolution of inflammation by releasing anti-inflammatory cytokines while dampening the secretion of pro-inflammatory cytokines. This underscores efferocytosis as a pivotal process in regulating inflammatory responses and facilitating tissue repair (9, 10).

Cardiovascular and cerebrovascular diseases, such as stroke and atherosclerosis, represent significant global health challenges characterized by altered blood flow dynamics and vascular morphological changes, coupled with inflammation. In this context, efferocytosis has emerged as a focal point in contemporary research aimed at understanding its mechanistic underpinnings and its impact on the pathophysiology of cardiovascular and cerebrovascular diseases (11, 12). Therefore, this review consolidates current insights into the mechanisms of efferocytosis and its implications for cardiovascular and cerebrovascular diseases.

## 2 Mechanisms of efferocytosis

**Figure 1:**
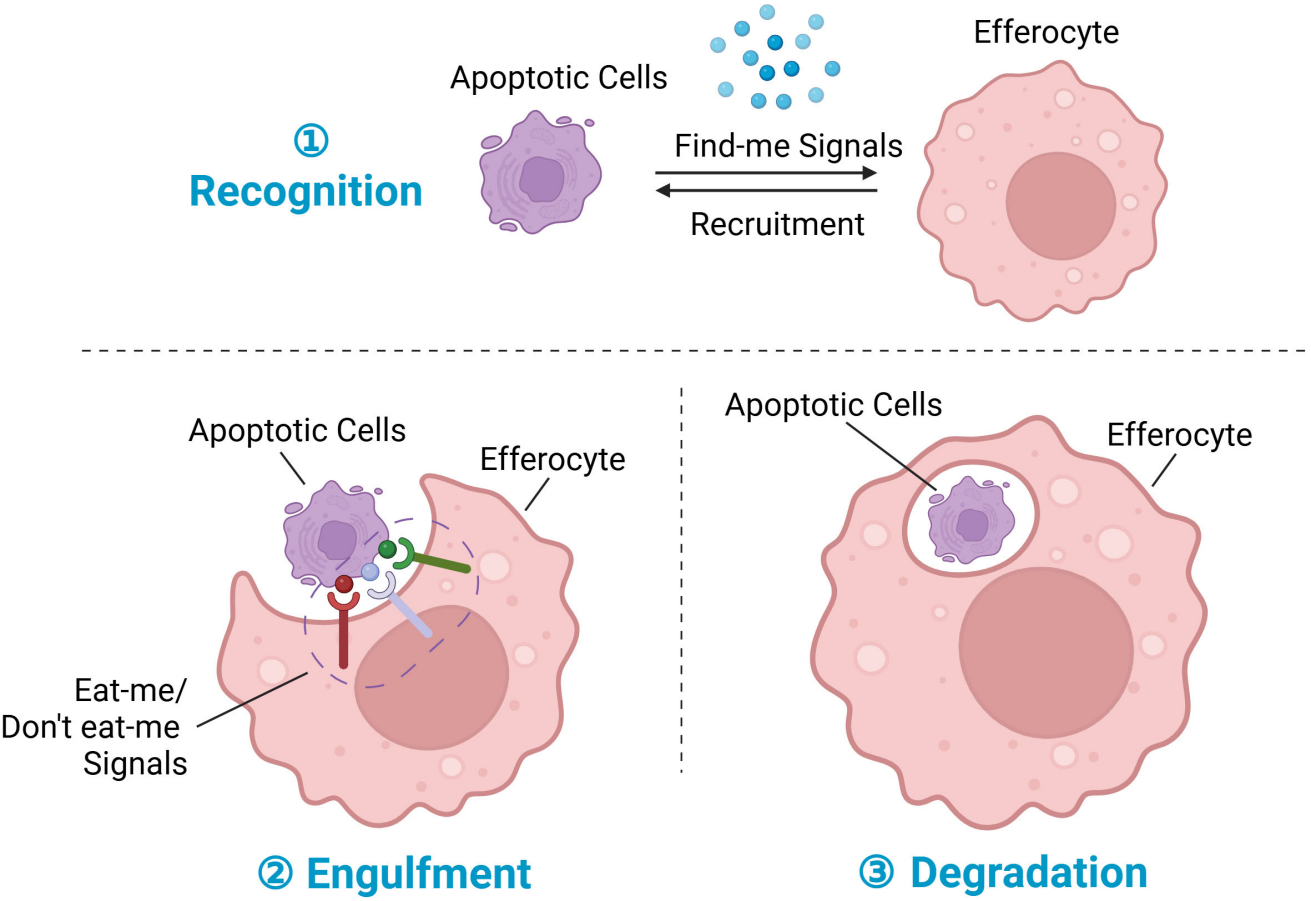
Steps in efferocytosis. ① Recognition phase: Following tissue injury, cells at the injury site undergo various programmed cell death pathways, notably apoptosis, ferroptosis, pyroptosis, and necroptosis, with apoptosis being the most prominent. During apoptosis, dying cells release chemokines termed “find-me” signals, which serve to recruit efferocytes to the injury site. ② Engulfment phase: Membrane proteins on dying cells interact with corresponding receptors on phagocytes, facilitating the regulation of efferocytosis; these membrane proteins are collectively referred to as “eat-me” signals or “don’t eat-me” signals. ③ Degradation phase: Upon recognition and engulfment of dying cells, the process of subsequent degradation commences. This process involves the maturation of the phagosome, its transformation into a phagolysosome, and the subsequent breakdown and digestion of the engulfed dying cells. Created with BioRender.com.

Efferocytosis plays a pivotal role in tissue repair and homeostasis by orchestrating the recognition, engulfment, and digestion of dying cells (**Figure 1**) (13). Optimal uptake of apoptotic cargo by phagocytes necessitates a finely tuned phagosomal machinery and requisite modifications. Following the recognition of apoptotic cells (ACs), phagocytes undergo a series of morphological and functional adaptations. These include the suppression of pro-inflammatory cytokine secretion, potentiation of anti-inflammatory cytokine production, acceleration of inflammation resolution, and facilitation of tissue repair processes. Efficient clearance of dying cells not only resolves inflammation but also contributes decisively to the restoration of tissue and organ homeostasis (14). Consequently, enhancing any of these facets presents potential avenues to improve efferocytosis and mitigate inflammatory responses. Subsequent sections will delve into the molecular intricacies and therapeutic strategies targeting each phase of efferocytosis.

### 2.1 Recognition of dying cells: “Find-me” signals and “Eat-me” signals

Following tissue injury, cells at the injury site undergo various programmed cell death pathways, notably apoptosis, ferroptosis, pyroptosis, and necroptosis, with apoptosis being the most prominent (15). During these programmed cell death pathways, dying cells release chemokines termed “Find-me” signals, which serve to attract phagocytes to the injury site. Additionally, membrane proteins on dying cells interact with corresponding receptors on phagocytes, facilitating the regulation of efferocytosis; these membrane proteins are collectively referred to as “Eat-me” signals (14).

**Figure 2:**
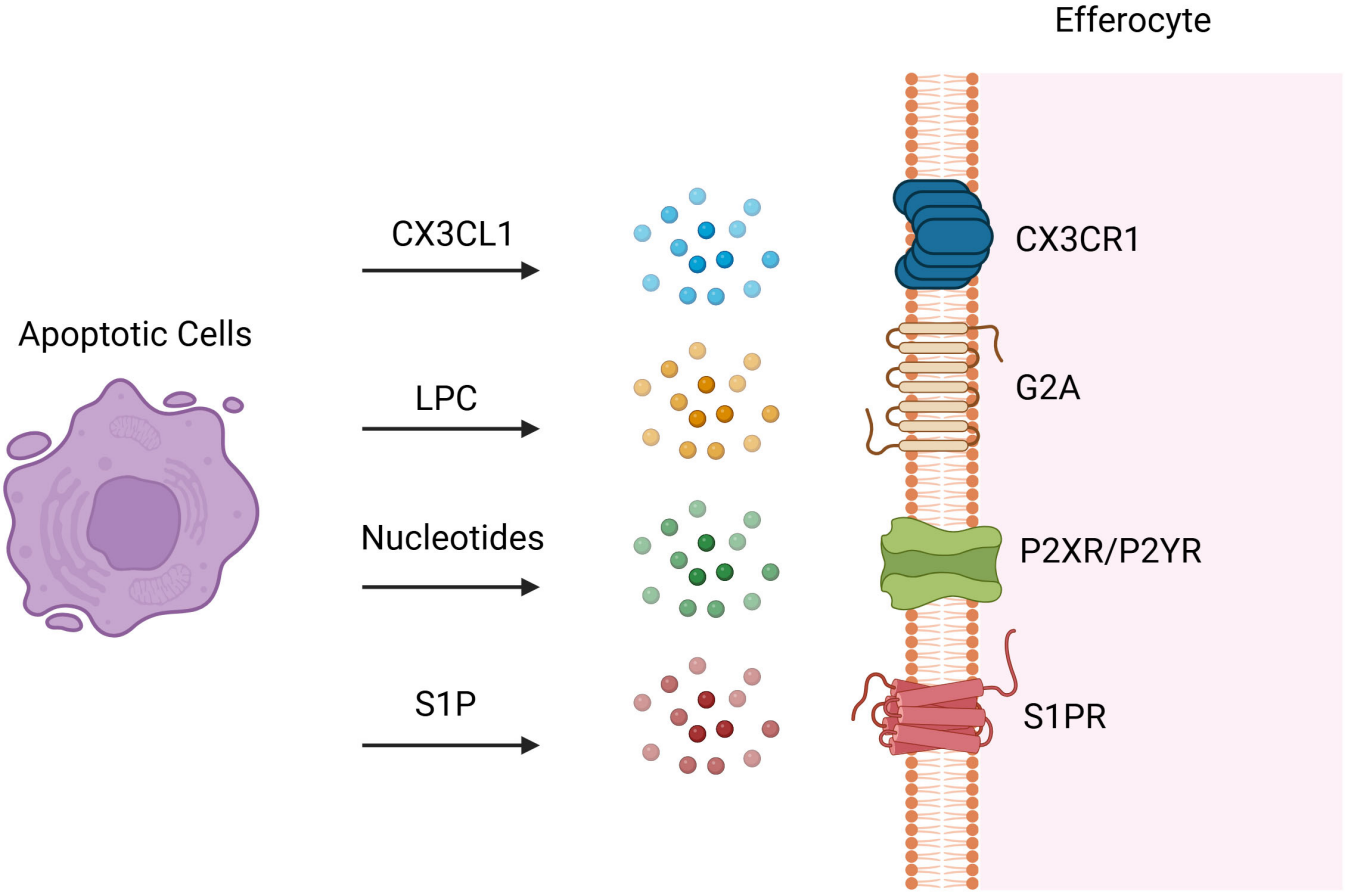
Find-me Signals. Following tissue injury, cells at the injury site undergo various programmed cell death pathways and dying cells release soluble find-me signals to attract efferocytes. These “find-me” signals encompass chemokines such as CX3C-chemokine ligand 1 (CX3CL1) also known as fractalkine, lipids such as sphingosine 1-phosphate (S1P) and lysophosphatidylcholine (LPC), and nucleotides such as ATP and UTP. Dying cells release CX3CL1, which binds to CX3CR1 on efferocytes and attract them to the injury site. LPC, an initial “find-me” signal, binds to G-protein-coupled receptor G2A to induce efferocytosis. S1P is synthesized in a caspase-dependent manner and abundantly released post-apoptosis and engages S1P receptors on efferocytes to induce efferocytosis. Nucleotides (ATP/UTP) also act as chemokines, promoting recruitment of efferocytes via binding to P2X or P2Y receptors. Created with BioRender.com.

After injury and apoptosis, cellular membranes undergo rupture and release soluble signals that function in dual capacities: firstly, as chemokines directing phagocytes towards apoptotic cells; and secondly, in priming phagocytes for engulfment by modulating their cytoskeletal dynamics and enhancing the expression of engulfment receptors and digestive machinery. These “find-me” signals encompass chemokines (e.g., CX_3_CL1 (16) also known as fractalkine), lipids [e.g., LPC (17)], and nucleotides (e.g., ATP and UTP). During early apoptosis, apoptotic cells release CX_3_CL1, which binds to CX_3_CR_1_ on phagocytes and guides them to the periphery of apoptotic cells (1). LPC, an initial “find-me” signal, binds to G-protein-coupled receptor G2A to induce efferocytosis, with ATP-binding cassette transporter 1 (ABCA1) expressed by apoptotic cells also facilitating LPC release (18). Sphingosine 1-phosphate (S1P), synthesized in a caspase-dependent manner, is abundantly released post-apoptosis and engages S1P receptors on phagocytes to promote efferocytosis (19, 20). Both LPC and S1P serve as apoptosis-specific “Find-me” signals. During apoptosis, caspase-3 cleavage activates calcium-independent phospholipase A2, which synthesizes LPC from phosphatidylcholine. Additionally, some apoptotic cells upregulate S1P mitogen-activated protein kinases SPK1 and SPK2, phosphorylating sphingosine to produce S1P (**Figure 2**) (21). Binding of S1P to S1P receptor (S1PR) on macrophages fosters an autocrine signaling loop involving hypoxia-inducible factor-1α (HIF-1α), erythropoietin (EPO), EPO receptor, and peroxisome proliferator-activated receptor-α (PPARα), which upregulates receptors for dying cells (22). Nucleotides (ATP/UTP) also act as chemokines, promoting recruitment of phagocytes via binding to P2X or P2Y receptors following cleavage of the C-terminal tail of Panx1 of plasma membrane Pannexin-1 (Panx1) channels by caspase 3/7 during apoptosis (6, 23). These released nucleotides “prime” phagocytes for engulfment by inducing expression of binding and engulfment receptors such as CD11b and α5β3 integrin (24).

**Figure 3:**
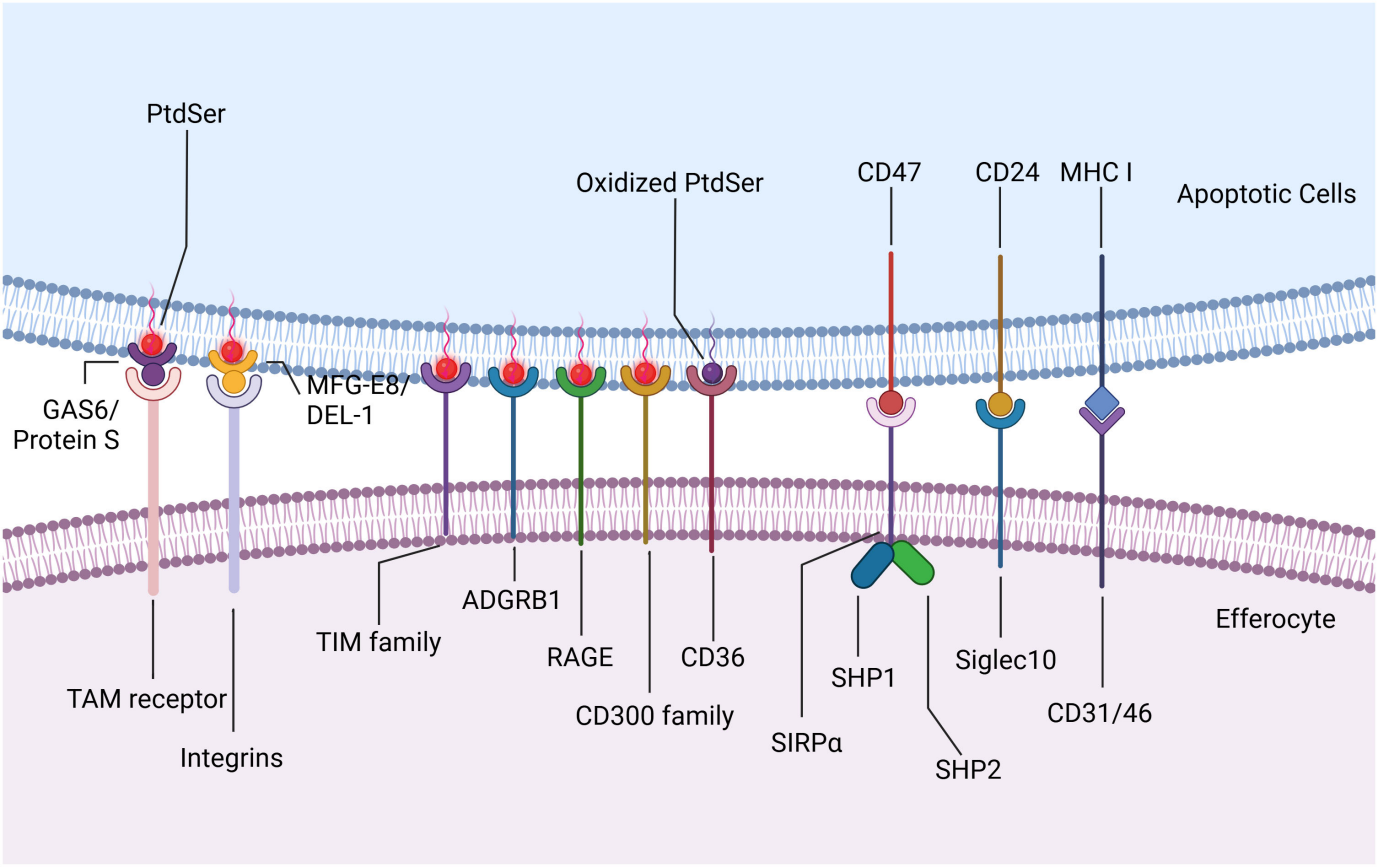
Eat-me Signals and Don’t eat-me Signals. Upon reaching the site of injury, efferocytes encounter “eat-me” signals displayed on the membranes of apoptotic cells, initiating downstream efferocytosis either directly or indirectly through receptor interactions. PtdSer translocates from the inner to the outer leaflet and exposes extracellularly for binding to efferocytes surface receptors directly. These receptors include stabilins such as stabilin1 and stabilin2, T cell immunoglobulin mucin receptors (TIM family) such as TIM1, TIM3, and TIM4, adhesion G protein-coupled receptor B1 (ADGRB1, also known as BAI1), the receptor for advanced glycation end products (RAGE), and members of the CD300 family such as CD300a and CD300b. Scavenger receptor CD36 interacts preferentially with oxidized PtdSer than non-oxidized PtdSer, enhancing phagocytic clearance of apoptotic cells. TAM receptor tyrosine kinases (TYRO3, AXL, and MerTK) recognize apoptotic cells via specific bridging ligands like growth arrest-specific protein 6 (GAS6) and Protein S. GAS6 interacts with all three TAM receptors to promote apoptotic cell binding, whereas Protein S only selectively binds to TYRO3 and MerTK. Integrins, another indirect receptor, require milk fat globule-EGF factor 8 (MFGE8) or its homologue developmental endothelial locus-1 (DEL-1) to bind PtdSer on apoptotic cells. In addition to “eat-me” signals, apoptotic cells also express “Don’t eat-me” signals to inhibit efferocytosis. CD47, typically expressed on healthy cells, interacts with SIRPα on phagocytes to prevent their engulfment. During apoptosis, CD47 on dying cells similarly engages with SIRPα, leading to the phosphorylation of SIRPα’s cytoplasmic domain and subsequent recruitment and activation of phosphatases SHP1/2. CD24 interacts with sialic acid binding immunoglobulin-like lectin 10 (Siglec10) on efferocytes, thereby evading efferocytosis. CD31, as well as CD46 with class I MHC molecules, contribute to preventing engulfment under healthy and apoptotic conditions. Created with BioRender.com.

Upon reaching the site of injury, phagocytes encounter “eat-me” signals displayed on the membranes of dying cells, initiating downstream efferocytosis either directly or indirectly through receptor interactions. These “eat-me” signals encompass phosphatidylserine (PtdSer), calreticulin, intercellular adhesion molecule 3 (ICAM3), and glycosylation moieties. In healthy cells, PtdSer is predominantly localized within the inner leaflet of plasma membranes, maintained by flippases. During apoptosis, caspase-3-mediated inactivation of flippases and activation of XKR8-independent scramblase activity induce rapid translocation of PtdSer from the inner to the outer leaflet, exposing it extracellularly for binding to phagocyte surface receptors directly (25). These receptors include stabilins (e.g., stabilin1 and stabilin2), T cell immunoglobulin mucin receptors (TIM family, such as TIM1, TIM3, and TIM4), adhesion G protein-coupled receptor B1 (ADGRB1, also known as BAI1), the receptor for advanced glycation end products (RAGE), and members of the CD300 family (CD300a and CD300b) (26, 27). Additionally, scavenger receptor CD36 interacts preferentially with oxidized PtdSer than non-oxidized PtdSer, enhancing phagocytic clearance of dying cells (28). Bridging ligands act as medium molecule between apoptotic cells and phagocytes, facilitating the binding of phagocytes to PtdSer indirectly. For instance, TAM receptor tyrosine kinases (TYRO3, AXL, and MerTK) recognize apoptotic cells via specific bridging ligands like growth arrest-specific protein 6 (GAS6) and Protein S. GAS6 interacts with all three TAM receptors to promote apoptotic cell binding, whereas Protein S only selectively binds to TYRO3 and MerTK (29). Integrins, another indirect receptor, require milk fat globule-EGF factor 8 (MFGE8) or its homologue developmental endothelial locus-1 (DEL-1) to bind PtdSer on dying cells (**Figure 3**) (30–32). Low-density lipoprotein receptor-related protein (LRP) can engage PtdSer with the aid of the bridging molecule beta-2-glycoprotein 1 (β2-GP1), facilitating engulfment by phagocytes (33). Other molecules acting as molecular bridges between apoptotic cargo and phagocytes include annexin A1 or lipocortin-1 interacting with PtdSer, and galectin-3 (Gal-3) interacting with MerTK (34, 35).

In addition to “eat-me” signals, dying cells also express “Don’t eat-me” signals such as CD47 and CD24. CD47, typically expressed on healthy cells, interacts with SIRPα on phagocytes to prevent their engulfment. During apoptosis, CD47 on dying cells similarly engages with SIRPα, leading to the phosphorylation of SIRPα’s cytoplasmic domain and subsequent recruitment and activation of phosphatases SHP1/2. These phosphatases inhibit phagocytosis by suppressing non-muscle myosin IIA activity (36, 37). CD24, recently identified as highly expressed on tumor cells, interacts with sialic acid binding immunoglobulin-like lectin 10 (Siglec10) on tumor-associated macrophages, thereby evading efferocytosis (38). Additionally, homophilic interactions of CD31 on leukocytes and macrophages, as well as CD46 with class I MHC molecules, contribute to preventing engulfment under healthy and apoptotic conditions (**Figure 3**) (39, 40). Although targeting these anti-phagocytic receptors is an underexplored area, it holds promise for influencing cardiovascular and cerebrovascular inflammation pathways.

### 2.2 Engulfment of dying cells

Efferocytosis is a tightly regulated process that encompasses the recognition, engulfment, and subsequent degradation of dead and dying cells. Upon recognition of dying cells by phagocytes, the process of engulfment and subsequent degradation commences. This process involves the maturation of the phagosome, its transformation into a phagolysosome, and the subsequent breakdown and digestion of the engulfed material.

#### 2.2.1 Uptake of dying cells

**Figure 4:**
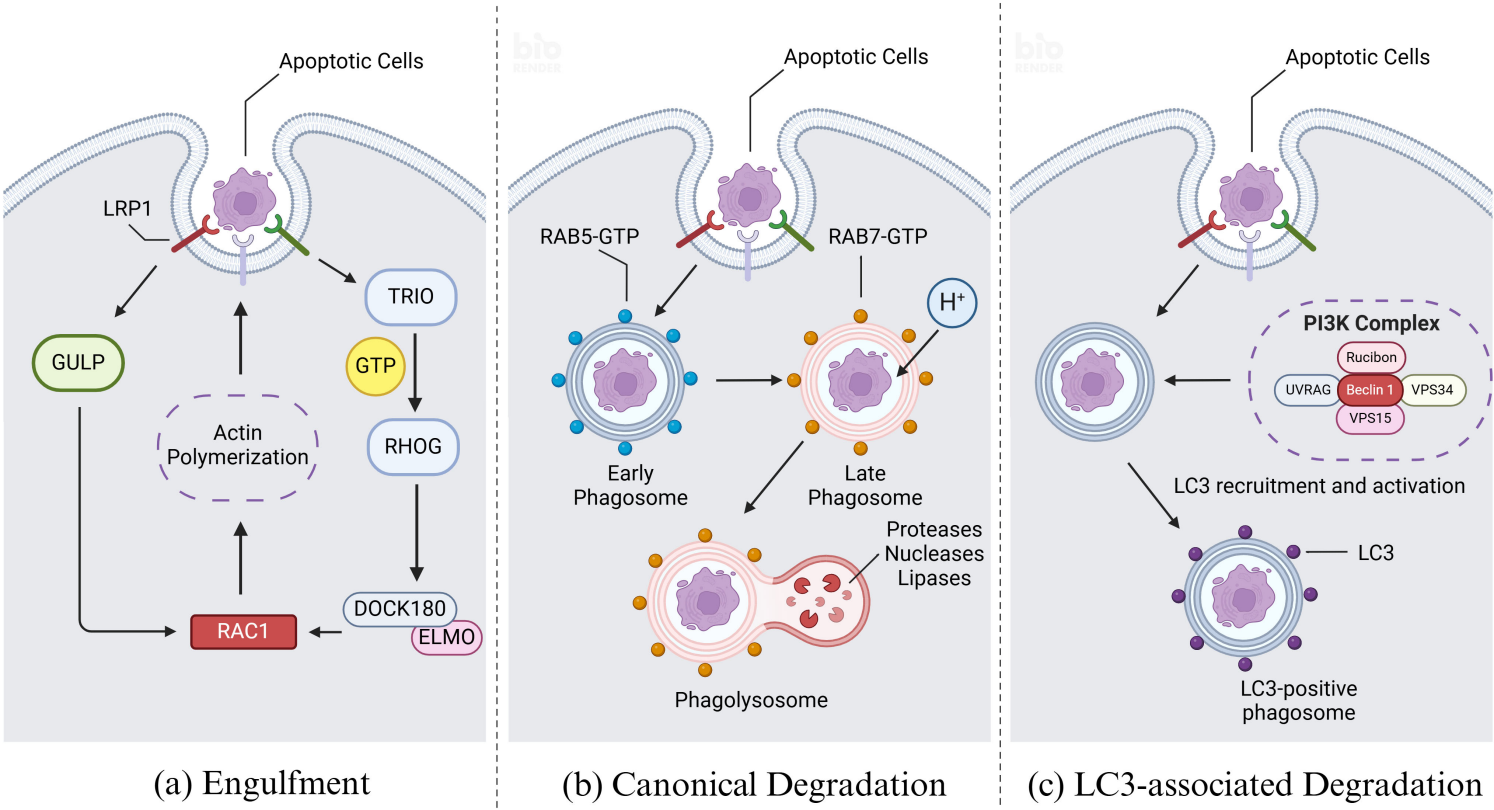
Engulfment and degradation of apoptotic cells. **(A)** Upon recognizing dying cells, efferocytes initiate actin remodeling and form phagosomes, ultimately leading to engulfment. Actin remodeling involves two primary mechanisms converging on the RHO family small GTPase RAC1. In the first mechanism, LRP1 and the adapter protein GULP are implicated in RAC1 activation, although the specific mechanism remains unclear. In the second mechanism, receptor ligation by apoptotic cells and efferocytes activates the guanine nucleotide exchange factor (GEF) ‘TRIO’, which loads GTP onto the small GTPase RHOG. This activation leads to recruitment of the engulfment and cell motility protein (ELMO), facilitating interaction with the SH3 domain of DOCK180. The resulting DOCK180-ELMO complex acts as another GEF for RAC1, thereby activating RAC1. Activated RAC1 then promotes the forming of the actin nucleation core which is necessary to coat or grasp the cargo. **(B)** Upon recognition of apoptotic cells by efferocytes, the membrane wraps around the apoptotic and enters the cell to form early phagosome. Afterwards the early phagosome matures and transforms into a late phagosome, distinguished by specific biochemical markers such as the acquisition of RAB7 and the concurrent loss of RAB5. Following the formation of the late phagosome, it undergoes fusion with lysosomes, which are rich in a diverse array of proteases, nucleases, and lipases responsible for the degradation of phagosomal cargo. Key to the regulation of these processes are the RAB GTPase family proteins, which cycle between an active, GTP-bound state and an inactive, GDP-bound state. RAB proteins interact with effector molecules that mediate various intracellular functions, including motor-driven vesicle trafficking, vesicle fusion events, and signaling pathways that regulate ‘RAB conversion’ and activation of downstream RAB GTPase family members. **(C)** Modification of phagosome maturation through LC3-associated phagocytosis (LAP) represents a noncanonical pathway involving autophagy proteins, specifically the microtubule-associated protein 1A/1B light chain 3 (LC3) family. Upon phagocytic engagement, a phosphatidylinositol 3-kinase (PI3K) complex assembles at the phagophore, comprising rubicon, UVRAG, beclin 1, VPS34, and VPS15. This complex phosphorylates phosphatidylinositides (PtdIns) to generate phosphatidylinositol 3-phosphate (PI3P), which is crucial for the activation of reactive oxygen species (ROS) production via NADPH oxidase-2 (NOX2). PI3P also facilitates the recruitment and activation of the LC3 conjugation machinery. Created with BioRender.com.

Upon recognizing dying cells, phagocytes initiate actin remodeling in the membrane and form phagosomes through alterations in membrane morphology, ultimately leading to engulfment (41). Actin remodeling involves two primary mechanisms converging on the RHO family small GTPase RAC1, a key regulator in this process. In one mechanism, LRP1 and the adapter protein GULP are implicated in RAC1 activation, although the specific mechanism remains unclear (42). In another mechanism, receptor ligation by dying cells and phagocytes activates the guanine nucleotide exchange factor (GEF) ‘TRIO’, which loads GTP onto the small GTPase RHOG. This activation leads to recruitment of the engulfment and cell motility protein (ELMO), facilitating interaction with the SH3 domain of DOCK180 (43). The resulting DOCK180-ELMO complex acts as another GEF for RAC1, thereby activating RAC1. Activated RAC1 then promotes localized actin polymerization, crucial for forming the actin nucleation core necessary to coat or grasp the cargo (**Figure 4A**) (44). This process involves nucleation-promoting factors from the WASP family, including SCAR and WAVE, which recruit the ARP2/3 complex to facilitate actin assembly (45).

#### 2.2.2 Degradation by lysosomes

Following the formation of the phagosome, it undergoes fusion with lysosomes, which are rich in a diverse array of proteases, nucleases, and lipases responsible for the degradation of phagosomal cargo (46). This fusion process is tightly regulated and influenced by multiple steps and biochemical changes at the phagosomal membrane. Key to the regulation of these processes are the RAB GTPase family proteins, which cycle between an active, GTP-bound state and an inactive, GDP-bound state. RAB proteins interact with effector molecules that mediate various intracellular functions, including motor-driven vesicle trafficking, vesicle fusion events, and signaling pathways that regulate ‘RAB conversion’ and activation of downstream RAB GTPase family members (**Figure 4B**) (47).

RAB5 plays a pivotal role in orchestrating phagosomes trafficking and the biogenesis of early phagosomes during maturation processes. It functions by recruiting and activating several critical effector proteins, including early endosomal antigen 1 (EEA1), the vacuolar fusion proteins MON1A and MON1B, and the class III phosphatidylinositol 3-kinase VPS34, all of which are essential for effective efferocytosis (48). Subsequently, VPS34 catalyzes the conversion of phosphatidylinositol into phosphatidylinositol 3-phosphate (PI3P), a key signaling lipid necessary for optimal phagosome maturation. This catalytic activity is further facilitated by the serine/threonine kinase VPS15, which forms a complex with VPS34 to activate RAB5 and promote its functions in phagosome biogenesis and trafficking (49, 50).

Early phagosomes undergo a transition to late phagosomes, distinguished by specific biochemical markers such as the acquisition of RAB7 and the concurrent loss of RAB5 (51). Late phagosomes further mature through the accumulation of two key RAB7 effector proteins: RAB7-interacting lysosomal protein (RILP) and oxysterol-binding protein-related protein 1 (ORP1 or ORPL1). These proteins facilitate interactions with the molecular motor dynein-dynactin, thereby coordinating microtubule-dependent vesicular trafficking of RAB7-positive late phagosomes (52). Concurrently, VAMP7 and syntaxin 7 are recruited to the phagosome membrane to form a Ca^2+^-dependent SNARE complex, promoting fusion between the phagosome and lysosome. Upon fusion, the resulting phagolysosome exhibits high acidity (pH 4.5-5.0), which is crucial for the efficient degradation of internalized cell corpse, facilitated by the presence of active cathepsins within the phagolysosomal lumen (53, 54).

### 2.3 Modification of phagosome maturation: LC3-associated phagocytosis

Modification of phagosome maturation through LC3-associated phagocytosis (LAP) represents a noncanonical pathway involving autophagy proteins, specifically the microtubule-associated protein 1A/1B light chain 3 (LC3) family (55). LC3 lipidation, critical for autophagosome trafficking and autophagosome/lysosome fusion, also plays a pivotal role in LAP, where the cargo primarily comprises dying cells and pathogens, distinct from the cellular organelles targeted in classical autophagy under intracellular stress conditions. LAP is particularly activated in response to injury or the phagocytosis of dying cells, in contrast to autophagy activation mechanism. Central to LAP is its regulatory function in innate immune cell activation subsequent to the recognition and engulfment of dead cell cargo (56). Upon phagocytic engagement, a phosphatidylinositol 3-kinase (PI3K) complex assembles at the phagophore, comprising rubicon, UVRAG, beclin 1, VPS34, and VPS15 (57). This complex phosphorylates phosphatidylinositides (PtdIns) to generate phosphatidylinositol 3-phosphate (PI3P), which is crucial for the activation of reactive oxygen species (ROS) production via NADPH oxidase-2 (NOX2). PI3P also facilitates the recruitment and activation of the LC3 conjugation machinery (**Figure 4C**). LC3 lipidation onto the phagosomal membrane promotes rapid phagosome maturation and subsequent phagosome-lysosome fusion, ensuring efficient clearance of dying cells and fostering an immune-silent environment (58).

## 3 Efferocytosis in cardiovascular disease

### 3.1 Atherosclerosis

Atherosclerotic cardiovascular diseases, such as atherosclerosis and myocardial infarction, result in more fatalities than all types of cancer combined. The principal pathophysiological mechanism involves the development of atherosclerotic plaques. These plaques originate from the accumulation of ApoB-containing lipoproteins within the subendothelial layer of arteries, initiating an inflammatory response that drives leukocyte influx into the vessel wall. Subsequently, these leukocytes undergo apoptosis and are cleared through efferocytosis. However, as the plaque advances, inefficient clearance of apoptotic cells leads to the accumulation of secondarily necrotic cells within the plaque, forming the necrotic core. These necrotic cores constitute the critical characteristic of plaque vulnerability, increasing the risk of rupture, luminal thrombosis, and thereby contributing significantly to the likelihood of myocardial infarction and stroke (2, 59). Hence, efferocytosis plays a pivotal role in resolving inflammation and mitigating atherosclerosis (**Table 1**).

**Table 1:** Targets involved in efferocytosis in cardiovascular disease.

Molecule/ Targets	Role in Efferocytosis	Ligands	Disease Model	Impact on Pathogenesis	Efferocytosis	Reference
MerTK↓	Eat-me	GAS6/Protein S	Ldlr^–/–^ mice Apoe^–/–^ mice	plaque↑	↓	(60, 61)
CAMKIIγ↑			Ldlr^–/–^ mice	plaque↑	↓	(62)
LRP1↓	Eat-me		Apoe^–/–^ mice	plaque↑	↓	(63, 64)
Tim-1/4↓	Eat-me		Ldlr^–/–^ mice	plaque↑	↓	(65)
SR-BI↓	Eat-me		Ldlr^–/–^ mice Apoe^–/–^ mice	plaque↑	↓	(66)
NLRP3↓			Ldlr^–/–^ mice Apoe^–/–^ mice	plaque↓	↑	(67)
PHACTR1↓			Apoe^–/–^ mice	plaque↓	↑	(68)
CNP↑			Apoe^–/–^ mice	plaque↓	↑	(69)
ALDH2↓			Apoe^–/–^ mice	plaque↑	↓	(70)
CD47↑	Don’t Eat-me		Apoe^–/–^ mice	plaque↑	↓	(71)
Resolvin D1↑			Ldlr^–/–^ mice	plaque↓	↑	(72)
PKM2↓			Ldlr^–/–^ mice	plaque↓	↑	(73)
IL-10↑			Ldlr^–/–^ mice	plaque↓	↑	(74)
Arg1↑			Ldlr^–/–^ mice	plaque↓	↑	(9)
Treg↑			Ldlr^–/–^ mice	plaque↓	↑	(75)
CFH↓			Ldlr^–/–^ mice	plaque↓	↑	(76)
MerTK↓	Eat-me	GAS6/Protein S	Permanent left coronary artery ligation	myocardial infarct size↑	↓	(77)
S100A9↓			Permanent left coronary artery ligation	myocardial infarct size↑	↓	(78)
Legumain↑			Experimental MI Surgery	myocardial infarct size↓	↑	(79)
Ndufs4↓			Permanent left coronary artery ligation	myocardial infarct size↑	↓	(80)
IL-10↑			Experimental MI Surgery	myocardial infarct size↓	↑	(81)
Sectm1a↓			Experimental MI/R Surgery	Ischemia/reperfusion-induced cardiac injury↑	↓	(82)
MerTK↓	Eat-me	GAS6/Protein S		Ischemia/reperfusion-induced cardiac injury↑	↓	(83)
CD47↓	Don’t Eat-me			Ischemia/reperfusion-induced cardiac injury↓	↑	(84)
MSCs↑				Ischemia/reperfusion-induced cardiac injury↓	↑	(85)

Abbreviations in order of appearance: MerTK, MER Tyrosine Kinase; CAMKIIγ, Calcium/Calmodulin Dependent Protein Kinase II Gamma; LRP1, LDL Receptor Related Protein 1; Tim-1/4, T Cell Immunoglobulin And Mucin Domain Containing 1/4; SR-BI, Scavenger Receptor Class B Member 1; NLRP3, NLR Family Pyrin Domain Containing 3; PHACTR1, Phosphatase And Actin Regulator 1; CNP, C-Type Natriuretic Peptide; ALDH2, Aldehyde Dehydrogenase 2 Family Member; CD47, Integrin Associated Protein; PKM2, Pyruvate kinase isozyme type M2; IL-10, Interleukin-10; Arg1, Arginase 1; Treg, Regulatory T cells; CFH, Complement Factor H; S100A9, S100 calcium binding protein A9; Legumain, Asparaginyl Endopeptidase; Ndufs4, NADH, NADH Dehydrogenase (Ubiquinone) Fe-S Protein 4; Sectm1a, Secreted And Transmembrane Protein 1; MSCs, Mesenchymal Stem Cells; GAS6, Growth Arrest Specific Protein 6; Ldlr, Low Density Lipoprotein Receptor; Apoe, Apolipoprotein E. The symbols "↑, ↓" in "Molecule/Targets" mean the expression of these targets were increased or decreased after ICH. The symbols in "Impact on Pathogenesis" mean the plaque size were increased or decreased. The symbols in "Efferocytosis" mean the efferocytosis after ICH were upregulated or downregulated.

As atherosclerosis progresses, persistent inflammatory stimuli lead to the cleavage of MerTK from the cell surface. This cleaved MerTK competes with cell surface MerTK on phagocytes for binding with GAS6 and protein S, thereby impairing efferocytosis within the lesion. Genetically engineered mice expressing cleavage-resistant MerTK exhibit enhanced efferocytosis and reduced propensity for developing necrotic cores compared to control mice (60, 86). Conversely, mice lacking or expressing an inactive form of MerTK demonstrate increased plaque size and expanded necrotic core area (61, 87). Similarly, LRP1, progressively downregulated during atherosclerosis, can also be rendered inactive through ADAM17-mediated proteolytic cleavage akin to MerTK (86). Macrophages in advanced lesions in both human and murine models upregulate calcium/calmodulin-dependent protein kinase IIγ (CaMKIIγ), which suppresses the ATF6-LXRα-MERTK pathway. Consequently, Western diet-fed Ldlr^−/−^ mice lacking myeloid CaMKIIγ exhibit elevated macrophage MerTK expression, improved efferocytosis, and reduced necrotic core area in atherosclerotic lesions (62). Loss of LRP1 in hematopoietic cells exacerbates atherosclerosis by impairing efferocytosis and increasing lesion area and necrotic core size in high-fat diet-fed atheroprone mice (63, 64). Additionally, blocking antibodies against TIM1 or TIM4, which serve as ‘Eat-me signals’, promote atherosclerosis progression by inhibiting efferocytosis within the lesion, leading to secondary accumulation of necrotic cells and heightened production of pro-inflammatory cytokines. Furthermore, TIM blockade induces substantial reductions in regulatory T cells in circulation, which otherwise stimulate lesional efferocytosis in an IL-10-dependent manner (65). Scavenger receptor class B member 1 (SRB1) facilitates efferocytosis via the SRC-PI3K-RAC1 pathway, and its deletion in macrophages results in defective efferocytosis, increased plaque size, larger necrotic core area, and heightened inflammation in atherosclerosis (66). Moreover, caspase 1 inhibitor VX765 could suppress NLRP3 inflammasome assembly and atherosclerosis progression by promoting mitophagy and efferocytosis (67).

CDKN2B, a key gene in atherosclerosis, exhibits reduced calreticulin levels in Cdkn2b^−/−^Apoe^−/−^ mice compared to controls, contributing to increased plaque size and necrotic core area under Western diet conditions. Notably, atherosclerotic plaques from patients often exhibit genetic variations at the 9p21 locus, including the CDKN2B gene, which correlate with reduced calreticulin levels and compromised efferocytosis, thereby indicating poorer prognosis (68). C-type natriuretic peptide (CNP) negatively correlates with coronary atherosclerosis burden in patients by promoting an anti-inflammatory macrophage phenotype, enhancing efferocytosis, and reducing foam cell formation and necroptosis (69). Several genome-wide association studies have linked intronic variants in phosphatase and actin regulator 1 (PHACTR1) on chromosome 6p24 with coronary artery disease (CAD) risk. The rs9349379 risk allele G, which lowers PHACTR1 expression in macrophages, may increase CAD risk via impaired efferocytosis, as PHACTR1 prevents myosin light chain (MLC) dephosphorylation necessary for apoptotic cell engulfment (68). Clinical studies highlight ALDH2 rs671 mutation as a common risk factor for atherosclerotic cardiovascular diseases, where macrophage ALDH2 stabilizes Rac2 to facilitate efferocytosis and reduce atherosclerosis development. Wild-type ALDH2 interacts directly with Rac2 to inhibit its degradation, whereas the rs671 mutant enhances Rac2 instability through increased K48-linked polyubiquitination at lysine 123 (70).

In atherosclerotic plaques, CD47 expression is significantly upregulated on apoptotic and necroptotic cells, contributing to defective efferocytosis. Administration of CD47-blocking antibodies reverses this defect, normalizes vascular tissue clearance, and mitigates atherosclerosis in various mouse models (71). The long non-coding RNA myocardial infarction-associated transcript (MIAT), elevated in atherosclerosis patients, interferes with miR-149-5p post-translational processing, thereby increasing CD47 levels. Genetic targeting of MIAT in Apoe^−/−^mice decreases CD47 expression, improves efferocytosis, and reduces plaque necrosis (88).

Efferocytosis induces alterations in lipid mediator production that are pivotal for inflammation resolution. Macrophages exposed to apoptotic neutrophils or neutrophil-derived microparticles exhibit heightened expression of specialized pro-resolving mediators (SPMs), such as lipoxin A4, resolvins D1, D2, and E2, derived from long-chain fatty acids (72, 89). Concurrently, there is a reduction in pro-inflammatory prostaglandins and leukotriene B4. Advanced human atherosclerotic lesions demonstrate decreased levels of SPMs compared to early-stage lesions, correlating with impaired efferocytosis. Treatment of mice with advanced atherosclerosis using resolving mediators enhances lesional efferocytosis. Conversely, elevated levels of proinflammatory lipid mediators in advanced atherosclerosis can impair efferocytosis. For instance, 12(S)-hydroxyeicosatetraenoic acid, derived from arachidonic acid and elevated in progressing atherosclerotic lesions and serum of individuals with coronary artery disease, inhibits efferocytosis in human monocyte-derived macrophages by activating RHOA, thereby blocking phagosome internalization. This impairment can be rectified by co-incubation with statins, which inhibit RHOA through the blockade of isoprenylation, a critical RHOA-activating modification (90). Macrophage efferocytosis is intricately linked to energy metabolism. Elevated levels of an enzyme regulating energy metabolism have been observed in macrophages from patients with atherosclerotic coronary artery disease. Conditional deletion of PKM2 in myeloid cells of Ldlr^-/-^ mice fed a high-fat Western diet for 14 weeks upregulated LRP-1 and enhanced macrophage efferocytosis, leading to regression of lesions in the entire aorta and aortic sinus. Depletion of PKM2 also suppressed MCP-1, IL-1β, and IL-12 expression, indicating inhibition of the macrophage proinflammatory phenotype (73).

Effective efferocytosis contributes to the synthesis of IL-10, TGF-β, and Arg-1 synthesis in atherosclerotic plaques. During efferocytosis, macrophage ornithine decarboxylase promotes the production of putrescine, which up-regulates IL-10 expression through the MerTK-MAPK-ERK1/2 pathway (74). IL-10, in turn, enhances efferocytosis in advanced atherosclerosis via the IL-10/Vav1/Rac1 pathway (91). Effective efferocytosis also induces the production of prostaglandin E2 (PGE2) via the CD36/ERK/Ptgs2/COX2 pathway, and PGE2 further stimulates TGF-β synthesis, which contributes to anti-inflammatory and pro-resolving properties (92). Additionally, effective efferocytosis up-regulates Arg-1 expression in macrophages, often associated with M2 polarization. Arg-1 metabolizes arginine and ornithine derived from apoptotic cells to produce putrescine, which enhances Dbl/Rac1-mediated engulfment of secondary apoptotic cells, thereby sustaining efferocytosis and promoting regression of atherosclerosis (9).

Adaptive immunity plays a pivotal role in the pathogenesis of atherosclerosis. Regulatory T cells (Tregs) are instrumental in attenuating proinflammatory responses and promoting resolution of inflammation. Single-cell RNA-sequencing of immune cells within atherosclerotic plaques has underscored the critical role of Tregs in enhancing efferocytosis and resolving atherosclerotic cardiovascular disease (75, 91). Cell-autonomous regulation of complement component C3 by complement factor H (CFH) modulates macrophage efferocytosis and impacts the progression of atherosclerosis. In a murine model of atherosclerosis, CFH deficiency has been shown to limit plaque necrosis in a manner dependent on complement C3. Specifically, deletion of CFH in monocyte-derived inflammatory macrophages leads to dysregulated cell-autonomous consumption of complement C3 without subsequent activation of downstream complement C5, thereby enhancing efferocytosis capacity (76).

### 3.2 Myocardial injury and repair

Heart failure subsequent to myocardial infarction (MI) continues to impose a significant burden of morbidity and mortality. Despite pharmacological advancements such as β-blockers and angiotensin-converting enzyme (ACE) inhibitors, which have effectively reduced mortality rates, the residual risk of developing heart failure post-MI remains considerable. Timely reperfusion through thrombolytic agents or primary percutaneous coronary intervention represents the most efficacious intervention for MI patients. However, reperfusion of the ischemic heart can induce additional damage, including extensive cardiomyocyte death and subsequent cardiac inflammation. This section will thus discuss recent research advances in efferocytosis in the context of MI (**Table 1**) (82, 93).

Following myocardial injury or ischemia/reperfusion injury, efferocytosis plays a critical role in the clearance of dying cells and damage-associated molecular patterns (DAMPs), thereby mitigating secondary cytokine storms and reducing inflammatory responses. Similar to its role in atherosclerosis, MerTK activation also facilitates efferocytosis in myocardial infarction. MerTK-mediated clearance of apoptotic cardiomyocytes accelerates inflammation resolution and enhances secretion of vascular endothelial growth factor A (VEGFA), thereby promoting cardiac repair post-injury (77). Maintaining elevated MerTK expression, potentially through extracellular vesicles secreted by cardiosphere-derived cells, could enhance efferocytosis efficacy and improve outcomes following myocardial infarction (94). Moreover, inhibiting the alarmin S100A9 may increase the population of reparative Ly6C^low^MerTK^high^ macrophages, thereby further enhancing efferocytosis and facilitating cardiac recovery (78).

As the principal phagocytes in the cardiovascular system, macrophages play a crucial role in efferocytosis following myocardial infarction. Cardiac resident macrophages, through the action of legumain, contribute to cardiac repair by facilitating the clearance and degradation of apoptotic cardiomyocytes post-myocardial infarction (79). Additionally, mitochondrial function in macrophages is pivotal in regulating cardiac repair processes after myocardial injury. Deletion of mitochondrial complex I protein Ndufs4 specifically in myeloid cells (mKO) recapitulates a proinflammatory metabolic phenotype in macrophages and exacerbates their response to lipopolysaccharide (LPS). Impaired efferocytosis in mKO macrophages results in reduced expression of anti-inflammatory cytokines and tissue repair factors, accompanied by heightened inflammatory responses. Mitochondria-targeted reactive oxygen species (ROS) scavenging ameliorates these impairments, enhances myofibroblast function, and reduces post-myocardial infarction mortality in mKO mice (80).

IL-10 is crucial in this context as an essential cytokine involved in MI. Co-incubation of apoptotic cells (ACs) with macrophages stimulates an increase in oxygen consumption rate and IL-10 production in macrophages, a process mediated by fatty acid oxidation. Fatty acids derived from apoptotic cells promote IL-10 synthesis through mitochondrial β-oxidation and regulation of electron transport, leading to an elevated NAD+/NADPH ratio. This heightened NAD+ level activates SIRT1, which in turn facilitates Pbx-1 binding to the apoptotic cell response element within the IL-10 promoter. Consequently, this cascade enhances IL-10 expression, thereby contributing to improved cardiac repair following ischemia/reperfusion injury (81).

Ischemia/reperfusion-induced cardiac injury represents a significant portion of myocardial damage. Macrophage-enriched Sectm1a plays a pivotal role in promoting effective efferocytosis to mitigate ischemia/reperfusion-induced cardiac injury and enhance cardiac function (82). Following ischemia/reperfusion-induced cardiac injury, MerTK undergoes cleavage similarly to the pattern observed in atherosclerosis in both humans and mice. The cleavage of MerTK on resident cardiac macrophages impairs efferocytosis and subsequent repair processes after myocardial ischemia/reperfusion injury (83). CD47 has been demonstrated to inhibit efferocytosis in the context of atherosclerosis. Consequently, genetically engineered macrophages co-loaded with CD47 inhibitors, such as PEP-20, could potentially synergistically restore efferocytosis and enhance cardiac remodeling following myocardial ischemia/reperfusion injury (84). Mesenchymal stem cells (MSCs) have shown considerable promise in the treatment of cardiovascular diseases, indicating their broad therapeutic potential. It has been established that MSC infusion can improve cardiac function in rats following myocardial ischemia/reperfusion, potentially through mechanisms that enhance M2 macrophage-mediated efferocytosis of apoptotic neutrophils (85).

## 4 Cerebrovascular disease

### 4.1 Intracerebral hemorrhage

Common cerebrovascular diseases in the population encompass ischemic stroke, intracerebral hemorrhage (ICH), and subarachnoid hemorrhage (SAH). Following the onset of these conditions, the efficient clearance of apoptotic cells is crucial for preserving central nervous system (CNS) homeostasis and facilitating recovery post-injury.

Intracerebral hemorrhage (ICH) is a severe condition characterized by hematoma-induced mass effect. Rupture of cerebral vessels leads to the accumulation of millions of red blood cells (RBCs) within the brain parenchyma, forming a hematoma. Surgical evacuation of hematoma is generally not recommended for most ICH cases due to uncertain clinical benefits and potential surgical complications. Hemolysis within the hematoma can generate toxic byproducts that contribute to significant secondary injuries and irreversible neurological deficits. Hence, promoting rapid hematoma clearance is crucial (95, 96).

Microglia and macrophages, acting as phagocytes, are swiftly recruited to the hemorrhage site to remove RBCs via erythrophagocytosis, a process vital for detoxifying hemolytic products and fostering neurological recovery post-ICH (**Table 2**) (120). Monocyte-derived macrophages (MDMs) exhibit heightened phagocytic activity and erythrophagocytosis within the ICH-afflicted brain. Recent research has discerned distinct roles of brain tissue-resident microglia and MDMs in the context of hemorrhagic brain injury. Initially, distinguishing between macrophages and microglia *in vivo* was challenging due to their shared origin and functional similarities. However, advancements in specific cell markers and multichannel flow cytometry have enabled researchers to differentiate these cell types. Consequently, researchers now refer to both microglia and macrophages collectively as Mφ (121).

**Table 2:** Targets involved in efferocytosis in cardiovascular disease.

Molecules/ Targets	Role in Efferocytosis	Ligands	Disease Model	Efferocytosis	Neuroinflammation	Neurological function	Reference
Axl↑	Eat-me	GAS6	Autologous blood-injection ICH model	↑	↓	↑	(97)
MerTK↑	Eat-me	GAS6/Protein S		↑	↓	↑	(86)
TLR-4↑				↓	↑	↓	(98)
CD36↑	Eat-me			↑	↓	↑	(99)
Soluble Trem2↑				↓	↑	↓	(100)
CD47↑	Don’t Eat-me			↓	↑	↓	(101, 102)
STAT6↓				↓	↑	↓	(103)
TNF-α↑				↓	↑	↓	(99)
IL-10↑				↑	↓	↑	(104, 105)
PPARγ↑				↑	↓	↑	(106)
Nrf2↑				↑	↓	↑	(107)
LRRC8A↑				↑	↓	↑	(108)
Irg1↓				↓	↑	↓	(109)
CBS↑				↑	↓	↑	(110)
Axl↑	Eat-me	GAS6	Transient MCAO model	↑	↓	↑	(111)
MerTK↑	Eat-me	GAS6/Protein S	BCAS model	↑	↓	↑	(112)
CD47↓	Don’t Eat-me		Transient MCAO model	↑	↓	↑	(113)
Trem2↓				↓	↑	↓	(114, 115)
P2Y6R↑				↑	↓	↑	(116)
Sig-1R↓				↓	↑	↓	(117)
CD300a↓				↑	↓	↑	(27)
STAT6↑				↑	↓	↑	(118)
C1q↑				↑	↓	↑	(7)
C3aR↑			Permanent MCAO model	↑	↓	↑	(119)

Abbreviations in order of appearance: Axl, Receptor Tyrosine Kinase; MerTK, MER Tyrosine Kinase; TLR-4, Toll-like Receptor-4; CD36, Cluster of Differentiation 36, Trem2, Triggering Receptor Expressed on Myeloid Cells 2; CD47, Integrin Associated Protein; STAT6, Recombinant Signal Transducer and Activator of Transcription 6; TNF-α, Tumor Necrosis Factor Alpha; IL-10, Interleukin-10; PPARγ, Peroxisome Proliferator-Activated Receptor Gamma; Nrf2, Nuclear factor erythroid 2-related factor 2; LRRC8A, Leucine Rich Repeat Containing 8 VRAC Subunit A; Irg1, Immune-Responsive Gene 1; CBS, Cystathionine β-Synthase; P2Y6R, Purinergic P2Y6 receptor; Sig-1R, Sigma-1 receptor; CD300a, Cluster of Differentiation 300A; C1q, Complement C1q; C3aR, Complement C3a Receptor. The symbols "↑, ↓" in "Molecule/Targets" mean the expression of these targets were increased or decreased after Ischemia stroke. The symbols in "Efferocytosis" mean the efferocytosis after Ischemia stroke were upregulated or downregulated. The symbols in "Neuroinflammation" mean the neuroinflammation after Ischemia stroke were promoted or mitigated. The symbols in "Neurological function" mean the neurological function after Ischemia stroke were improved or reduced.

Tyro3, predominantly expressed on neurons rather than Mφ within the central nervous system (CNS), contrasts with Axl and MerTK, which are primarily expressed on Mφ. In a murine model, the transcriptional levels of Axl, MerTK, and their ligand Gas6 increase within 24 hours post-intracerebral hemorrhage (ICH), and deficiency of Axl/MerTK impairs macrophage-mediated erythrophagocytosis in ICH (97). Toll-like receptors (TLRs) induce a pro-inflammatory Mφ phenotype (M1), whereas Axl/MerTK activation suppresses TLR signaling through suppressors such as SOC1 and SOC3, promoting an anti-inflammatory Mφ phenotype (M2) (98, 122). Exogenous ligands, including recombinant Gas6, can target Axl/MerTK-mediated erythrophagocytosis akin to approaches used in atherosclerosis. Following ICH, Axl/Mertk undergo cleavage from the cell membrane, generating soluble but non-functional forms (sAxl/sMertk). These soluble forms competitively bind to endogenous ligands (Gas6 and Pros1), thereby depleting ligands crucial for maintaining homeostasis. Consequently, exogenous recombinant Gas6 enhances efferocytosis and resolves inflammation in an Axl-dependent manner following ICH (86, 97).

CD36, recognized as an ‘eat-me’ signal, exhibits elevated transcription in microglia cultures treated with erythrocytes and within the perihematomal region following intracerebral hemorrhage (ICH) (123, 124). Genetic deletion or antibody blockade of CD36 impedes erythrocyte phagocytosis by microglia. CD36 knockout mice demonstrate delayed hematoma resolution and exacerbated deficits compared to wild-type (WT) mice post-ICH. Patients lacking CD36 exhibit impaired hematoma resolution and poorer clinical outcomes. Upregulation of CD36 accelerates erythrophagocytosis and enhances hematoma resolution (99). Soluble Trem2 has been shown to negatively regulate erythrophagocytosis post-ICH via CD36 receptor recycling, mediated by reduced VPS35 (100). Similarly, CD47, ubiquitously expressed across various cell types including erythrocytes, serves as a ‘don’t eat me’ signal by binding to signal regulatory protein α (SIRPα) on macrophages (Mφ) to inhibit erythrophagocytosis. Perihematomal levels of CD47 initially rise within hours post-ICH but subsequently decline, coinciding with Mφ infiltration and erythrophagocytosis (101, 125). Intracranial injection of CD47 knockout blood accelerates hematoma resolution and reduces brain edema, effects attenuated by intracranial clodronate liposome administration, a specific Mφ depletion agent (102). Furthermore, CD47 blocking antibodies significantly enhance erythrophagocytosis and promote hematoma clearance following ICH (126, 127).

Interleukin-4 (IL-4) serves as a canonical activator of signal transducer and activator of transcription 6 (STAT6), and exogenous IL-4 administration has been shown to activate STAT6 and enhance erythrophagocytosis in intracerebral hemorrhage (ICH). STAT6 knockout (KO) mice demonstrate exacerbated outcomes compared to wild-type (WT) counterparts in ICH models, showing reduced responsiveness to IL-4 treatment and impaired phagocytic capacity of red blood cells by phagocytes (103). Transcriptomic analyses revealed diminished expression of IL-1 receptor-like 1 (ST2) in microglia/macrophages of STAT6 KO mice post-ICH, underscoring the significance of IL-4/STAT6/ST2 signaling in hematoma resolution and functional recovery after ICH (128). Intranasal IL-4 treatment warrants further investigation as a potential therapeutic strategy for ICH. Moreover, the IL-4/STAT6 axis has been observed to upregulate CD36, a scavenger receptor critical for initiating efferocytosis, potentially through direct binding of STAT6 to the promoter regions of the CD36 gene (129). IL-4/STAT6 signaling also transcriptionally upregulates anti-inflammatory cytokines such as Arg1, which are essential for STAT6-mediated pro-phagocytic activity in Mφ and sustained efferocytosis (118). Conversely, TNF-α inhibits apoptotic cell clearance and polarizes Mφ towards a proinflammatory phenotype, concurrently downregulating CD36 expression in microglia and impairing erythrophagocytosis, while also upregulating the ‘don’t eat-me’ signal CD47 in vascular smooth muscle cells, further hindering erythrophagocytosis (99). IL-10, known for its anti-inflammatory properties, has been reported to promote erythrophagocytosis by regulating CD36 expression (104). Additionally, IL-10 delivery via phosphatidylserine liposomes has shown promise in improving erythrophagocytosis and clinical outcomes in ICH (105).

Transcription factors within Mφ are pivotal in regulating erythrophagocytosis and recovery following intracerebral hemorrhage (ICH). PPARγ transcriptionally upregulates critical scavenger receptors such as Axl, MerTK, and CD36, which are essential for erythrophagocytosis (106, 124). Nrf2 serves as a principal transcription factor protecting cells against endogenous and exogenous stressors, and its activation has been demonstrated to enhance erythrophagocytosis by upregulating CD36 (107). Both PPAR γ and Nrf2 also modulate the NF-κB pathway, contributing to inflammation attenuation in ICH, thereby highlighting targeting PPAR γ and Nrf2 as promising strategies to augment hematoma resolution (130). Bexarotene, an FDA-approved selective RXR (retinoid X receptor) agonist used clinically in cutaneous T-cell lymphoma, enhances the expression of PPARγ-dependent genes through RXR heterodimerization, thereby improving erythrophagocytosis and recovery in ICH (131). Recent insights underscore the regulation of Mφ phagocytosis via the LRRC8A channel through the AMPK-Nrf2-CD36 pathway following ICH, suggesting LRRC8A as a potential therapeutic target for enhancing hematoma clearance (108). Itaconate, an intermediate of the tricarboxylic acid cycle, is produced from the decarboxylation of cis-aconitate by immune-responsive gene 1 (Irg1) within mitochondria. Depletion of Irg1 in macrophages/microglia diminishes erythrocyte phagocytosis, exacerbating outcomes in ICH. Administration of sodium itaconate or 4-octyl itaconate (4-OI) promotes macrophage phagocytosis via the Keap1-Nrf2-CD36 pathway (109). The Irg1/itaconate axis represents a potential therapeutic target for disorders characterized by phagocytosis deficiency, such as ICH.

In addition to the aforementioned findings, hydrogen sulfide (H2S), a gasotransmitter, acts as an endogenous regulator facilitating sustained phagocytosis following intracerebral hemorrhage (ICH). Expression of the H2S synthase cystathionine β-synthase (CBS) and CBS-derived H2S is upregulated in brain-resident phagocytic microglia post-ICH, thereby enhancing continuous erythrocyte phagocytosis via the CBS-H2S-complex I axis (110). Fan et al. have developed pH-responsive pro-efferocytic nanoparticles resembling neutrophils, designed to enhance neurological recovery by promoting erythrophagocytosis after ICH (132).

### 4.2 Ischemic stroke

Ischemic stroke represents a significant public health burden and remains the leading cause of mortality and disability worldwide. Apart from thrombolysis and thrombectomy during the acute stage, effective therapeutic strategies remain limited (133). The disruption of regional blood supply initiates an ischemic cascade that results in neuronal dysfunction and subsequent cell death. During the sub-acute phase, brain edema and inflammatory responses further contribute to secondary injury processes (134).Following ischemic stroke onset, microglia and macrophages undergo rapid activation and recruitment to the infarct site, promoting efferocytosis (**Table 2**). TAM receptors, including Axl and MerTK, play crucial roles in enhancing efferocytosis post-ischemic stroke (111, 112). Phagocytic processes are initiated by exposure of “eat-me” signals on target cells or debris, such as phosphatidylserine (PS) exposed on the membranes of dying cells, which interacts with MFG-E8, Axl, and MerTK to facilitate efferocytosis and mitigate injury. Additionally, the ligands Gas6 and Protein S are involved in mediating these processes after ischemic stroke (135). Conversely, ‘don’t eat-me’ signals can modulate microglia and macrophage efferocytosis during ischemic stroke. Research utilizing CD47 knockout mice indicates that deletion of CD47 reduces brain infarction and edema during the acute phase in the middle cerebral artery occlusion (MCAO) model by mitigating neuroinflammation (113).

Accumulating evidence underscores the significant impact of the Triggering receptor expressed on myeloid cells-2 (TREM2)-activating protein of 12kDa (DAP12) system in central nervous system CNS disorders such as neurodegenerative diseases and stroke (136). TREM2, initially expressed on macrophages and microglia, relies on DAP12 as an intracellular membrane adaptor (137). Microglial deficiency in TREM2 impedes the clearance of apoptotic neurons and exacerbates the production of inflammatory cytokines like TNF-α (138). Moreover, TREM2 facilitates efferocytosis of dying cells following experimental stroke. Deficiency in TREM2 exacerbates outcomes after ischemic stroke by diminishing efferocytosis of dying neurons and microglia, underscoring its pivotal role over circulating macrophages in ischemic conditions (114). Furthermore, a high-salt diet has been shown to reduce the efferocytic capacity of macrophages by downregulating TREM2 expression, thereby hindering the resolution of neuroinflammation post-ischemic stroke (139). Augmenting TREM2 signaling in monocytes/macrophages represents a promising therapeutic approach to enhance efferocytosis and promote inflammation resolution following stroke. Recent studies employing microscale thermophoresis (MST), surface plasmon resonance (SPR), and liquid chromatography-tandem mass spectrometry (LC-MS/MS) have revealed sphingosine-1-phosphate (S1P) as a novel ligand for TREM2, which targets the TREM2-DAP12 complex to promote microglial efferocytosis and protect against ischemic brain injury (115).

P2 purinoceptors play a significant role in the pathogenesis of ischemic stroke. These receptors are categorized into two families: ionotropic receptors (P2X) and metabotropic receptors (P2Y). The P2Y6 receptor expressed on microglia is activated by UDP released from dying neurons, initiating microglial efferocytosis to clear apoptotic cells. Upon activation by UDP, P2Y6 receptors induce actin cytoskeleton rearrangement, forming filopodia-like protrusions that facilitate the engulfment of cellular debris (140). Expression of P2Y6 receptors in microglia increases following transient middle cerebral artery occlusion (tMCAO), and inhibition of P2Y6 receptors with the antagonist MRS2578 suppresses microglial phagocytosis of cell debris, thereby exacerbating neurological deficits (116). In addition to the P2Y6/UDP signaling pathway, neuronal injury leads to ATP or ADP release, which recruits microglia to the site of injury through P2Y12 receptors (141). Post-ischemic stroke, P2Y12-mediated chemotaxis of microglia plays a critical role in maintaining blood-brain barrier (BBB) integrity (142). Sigma-1 receptor (Sig-1R) is a chaperone protein that modulates diverse cellular functions, including cell death, autophagy, apoptosis, neuronal differentiation, and neuroinflammation. Depletion of Sig-1R markedly impairs the phagocytic activity of macrophages and microglia, resulting in exacerbated brain damage and neurological deficits. Mechanistically, Sig-1R-mediated efferocytosis relies on Rac1 activation, and specific binding pockets responsible for Sig-1R interactions have been identified (117). CD300a, has a long cytoplasmic region that contains immunoreceptor tyrosine-based inhibitory motifs (ITIMs) akin to SIRPα, also enhances efferocytosis by infiltrating myeloid cells and ameliorates neuronal deficit after ischemic stroke via blocking by its neutralizing antibody (27).

Macrophages play a pivotal role in the phagocytic clearance of deceased neurons following ischemic stroke. Robust transcriptomic alterations occur in monocytes/macrophages infiltrating the post-stroke brain. These changes include significant upregulation of numerous efferocytosis-related genes within brain macrophages. Transcriptomic analyses further reveal that PPARγ and STAT6 act as potential upstream regulators that shape the pro-efferocytic and inflammation-resolving transcriptome of macrophages in the post-stroke brain, akin to their roles observed in intracerebral hemorrhage (ICH) (143). Members of the STAT family are critical in regulating the functional state of microglia/macrophages, with the STAT6/Arg1 axis identified as a key signaling mechanism for phenotypic modulation in the context of ischemic stroke. Activation of the STAT6/Arg1 axis promotes efferocytosis by microglia/macrophages and contributes to inflammation resolution in mouse models of stroke (118).

In addition to the previously discussed non-specific and standard receptors, adaptive immunity also plays a pivotal role in the pathogenesis of ischemia stroke. Complement components C1q and C3 play significant roles in inducing efferocytosis through their interaction with dying cell surfaces. C1q is widely distributed in the central nervous system (CNS), including neutrophils, microglia, and a subset of interneurons. Following ischemic stroke, activation of the complement system leads to increased C1q levels, which enhance microglial efferocytosis by binding to apoptotic cells and neuronal blebs, thereby protecting the CNS (7). C3 is activated and cleaved by C3 convertase into C3a, a small protein that recruits immunocytes and modulates immune responses through interaction with its receptor, C3aR (144). Intracortical administration of the complement C3 receptor antagonist trifluoroacetate has been shown to modulate microglial responses and attenuate neuronal death following brain injury (119). C3b, a product of C3 cleavage, facilitates the clearance of dying cells and modulates adaptive immune responses. Modification of glycoproteins or glycolipids represents a prominent strategy to regulate efferocytosis induced by C1q and C3. Desialylation of glycoproteins or glycolipids enhances recognition by C1q, thereby promoting efferocytosis. Conversely, sialic acid modification of glycoproteins or glycolipids acts as a ‘don’t eat-me’ signal by preventing binding of complement C1q and C3b, thereby modulating the clearance of dying cells (145).

### 4.3 Subarachnoid hemorrhage

Subarachnoid hemorrhage (SAH) is a relatively common cause of stroke, with an annual incidence of approximately 6-7 cases per 100,000 individuals, predominantly affecting those under 55 years of age. Despite accounting for only 5% of all stroke cases, SAH exhibits a high initial mortality rate, reaching up to 67% in the first few months. Recent studies have highlighted the crucial role of efferocytosis in early brain injury (EBI) following SAH (146). SAH triggers the upregulation of Axl and its ligand Gas6. Administration of recombinant Gas6 (rGas6) has been shown to enhance efferocytosis, reduce inflammation, and mitigate SAH-induced blood-brain barrier (BBB) breakdown and neurological deficits through the Axl/Rac1 signaling pathway (97). Although the influence of other efferocytosis-related molecules on EBI after SAH remains less explored, many of these molecules are known to regulate neuroinflammation, which can be alleviated through efferocytosis (147). Consequently, they represent potential targets for future therapeutic interventions.

## 5 Concluding remarks

Cardiovascular and cerebrovascular diseases impose significant burdens on both patients and society owing to their unfavorable prognostic outcomes. Inflammation assumes a pivotal role in these conditions, whereas efferocytosis represents a potent mechanism for resolving inflammation and maintaining homeostasis. In recent years, scholarly attention has been directed towards investigating the mechanisms and exploring drug discovery avenues related to efferocytosis in cardiovascular and cerebrovascular diseases. Despite considerable advancements, significant challenges persist in developing methods that substantially enhance the prognosis of patients afflicted with cardiovascular and cerebrovascular disease. Nevertheless, efferocytosis and its underlying mechanisms continue to constitute a pivotal area for future research, offering potential to markedly improve the prognosis of patients with cardiovascular and cerebrovascular disease.
